# THE IMPORTANCE OF HSP-25 IN CAENORHABDITIS ELEGANS LONGEVITY

**DOI:** 10.1093/geroni/igz038.3176

**Published:** 2019-11-08

**Authors:** Niaya James, Jessica L Scheirer, Karl Rodriguez

**Affiliations:** 1 Howard University, washington, District of Columbia, United States; 2 UT Health Science Center, San Antionio, San Antionio, Texas, United States; 3 Sam and Ann Barshop Institute for Longevity and Aging Research, San Antonio, Texas, United States

## Abstract

Karl A. Rodriguez’s laboratory at the University of Texas Health Science Center, San Antonio, Texas, is interested in the role of small heat shock proteins in the proteostasis network and aging using the model organism, Caenorhabditis elegans. Molecular chaperones facilitate protein folding and improve the degradation activity of the proteasome and autolysosome hence decreasing disease-associated aggregates. Previous work in rodents have shown an increase in expression levels of the small heat shock protein 25 (HSP-25) correlates with maximum lifespan potential. To further explore the role of HSP-25 in C. elegans, two HSP-25 knock-out strains were exposed to a one-hour heat stress, heat shock, and two non-heat stress conditions.

